# A comparative study of mesenchymal stem cells cultured as cell‐only aggregates and in encapsulated hydrogels

**DOI:** 10.1002/term.3257

**Published:** 2021-10-22

**Authors:** Fiona R. Passanha, David B. Gomes, Justyna Piotrowska, Students of PRO3011, Lorenzo Moroni, Matthew B. Baker, Vanessa L. S. LaPointe

**Affiliations:** ^1^ Department of Cell Biology–Inspired Tissue Engineering MERLN Institute for Technology‐ Inspired Regenerative Medicine Maastricht University Maastricht The Netherlands; ^2^ Department of Complex Tissue Regeneration MERLN Institute for Technology‐Inspired Regenerative Medicine Maastricht University Maastricht The Netherlands; ^3^ University College Maastricht Maastricht University Maastricht The Netherlands

**Keywords:** 3D cell culture, alginate hydrogels, regenerative medicine

## Abstract

There is increasing evidence that cells cultured in three‐dimensional (3D) settings have superior performance compared to their traditional counterparts in monolayers. This has been attributed to cell–cell and cell–extracellular matrix interactions that more closely resemble the in vivo tissue architecture. The rapid adoption of 3D cell culture systems as experimental tools for diverse applications has not always been matched by an improved understanding of cell behavior in different 3D environments. Here, we studied human mesenchymal stem/stromal cells (hMSCs) as scaffold‐free self‐assembled aggregates of low and high cell number and compared them to cell‐laden alginate hydrogels with and without arginine‐glycine‐aspartic acid peptides. We observed a significant decrease in the size of cell‐only aggregates over 14 days in culture compared to the cells encapsulated in alginate hydrogels. Alginate hydrogels had persistently more living cells for a longer period (14 days) in culture as measured by total DNA content. Proliferation studies revealed that a weeklong culture of hMSCs in 3D culture, whether as aggregates or cell‐laden alginate hydrogels, reduced their proliferation over time. Cell cycle analysis found no significant differences between days 1 and 7 for the different culture systems. The findings of this study improve our understanding of how aggregate cultures differ with or without a hydrogel carrier, and whether aggregation itself is important when it comes to the 3D culture of hMSCs.

## 1 INTRODUCTION

Therapies to repair or regenerate damaged tissue by the transplantation of stem cells are a promising approach in the field of regenerative medicine. Mesenchymal stem/stromal cells (MSCs) are one such candidate because of their ability to differentiate into various cell types, their immunomodulatory properties, their capacity to migrate to the site of injury, their low risk of teratoma formation, and that they can be derived from many (autologous) tissues (Chung & Burdick, 2009; Galipeau & Sensébé, 2018; Gattazzo et al., 2014; Mendicino et al., 2014). Mesenchymal stem/stromal cell–based therapies have shown efficacy in treating patients with musculoskeletal injuries and disease, acute lung injury, traumatic brain injury, acute renal failure, cardiac injury, and other indications (Bruno et al., 2012; Iijima et al., 2018; Matthay et al., 2019; Walker et al., 2010). There are currently >20 ongoing phase 3 trials using MSCs (https://www.clinicaltrials.gov/), making it reasonable to expect that more therapies will be available to patients in the near future.

To date, the MSC field continues to struggle with how to best direct the behavior of the cells, and scientists are increasingly moving towards three‐dimensional (3D) culture to overcome this hurdle. In general, MSCs are reported to have improved behavior in 3D environments compared to monolayers. For example, spheroids of MSCs have higher osteogenic potential compared to cells in a monolayer both in vitro and in vivo (Yamaguchi et al., 2014). Similarly, they also induce enhanced chondrogenic differentiation by an increased expression of TGFβ3 (Yoon et al., 2012). Mesenchymal stem/stromal cell aggregates also secrete substantial quantities of potent anti‐inflammatory proteins compared to monolayer cells (Bartosh et al., 2010) and late passage MSCs cultured as spheroids can regain their immune‐modulatory factors (Bartosh & Ylostalo, 2019). The positive effects of 3D culture were also seen when medium conditioned by MSC spheroids effectively stimulated endothelial cell migration and proliferation compared to the medium conditioned by an adherent monolayer (Potapova et al., 2007).

There are multiple ways to confer a 3D environment onto MSCs. For example, they can self‐assemble into aggregates, be suspended in hydrogels, or combinations thereof. In all cases, positive effects on cell behavior have been reported; for example, embedding cells within 3D microenvironments such as alginate hydrogels has also been shown to improve their survival and also allow the secretion of endogenous healing factors (Grigore et al., 2014; Lee et al., 2019; Schmitt et al., 2015). However, there have been few direct comparisons that would provide insight into how the behavior of MSCs is affected by the different 3D culture systems.

**FIGURE 1:**
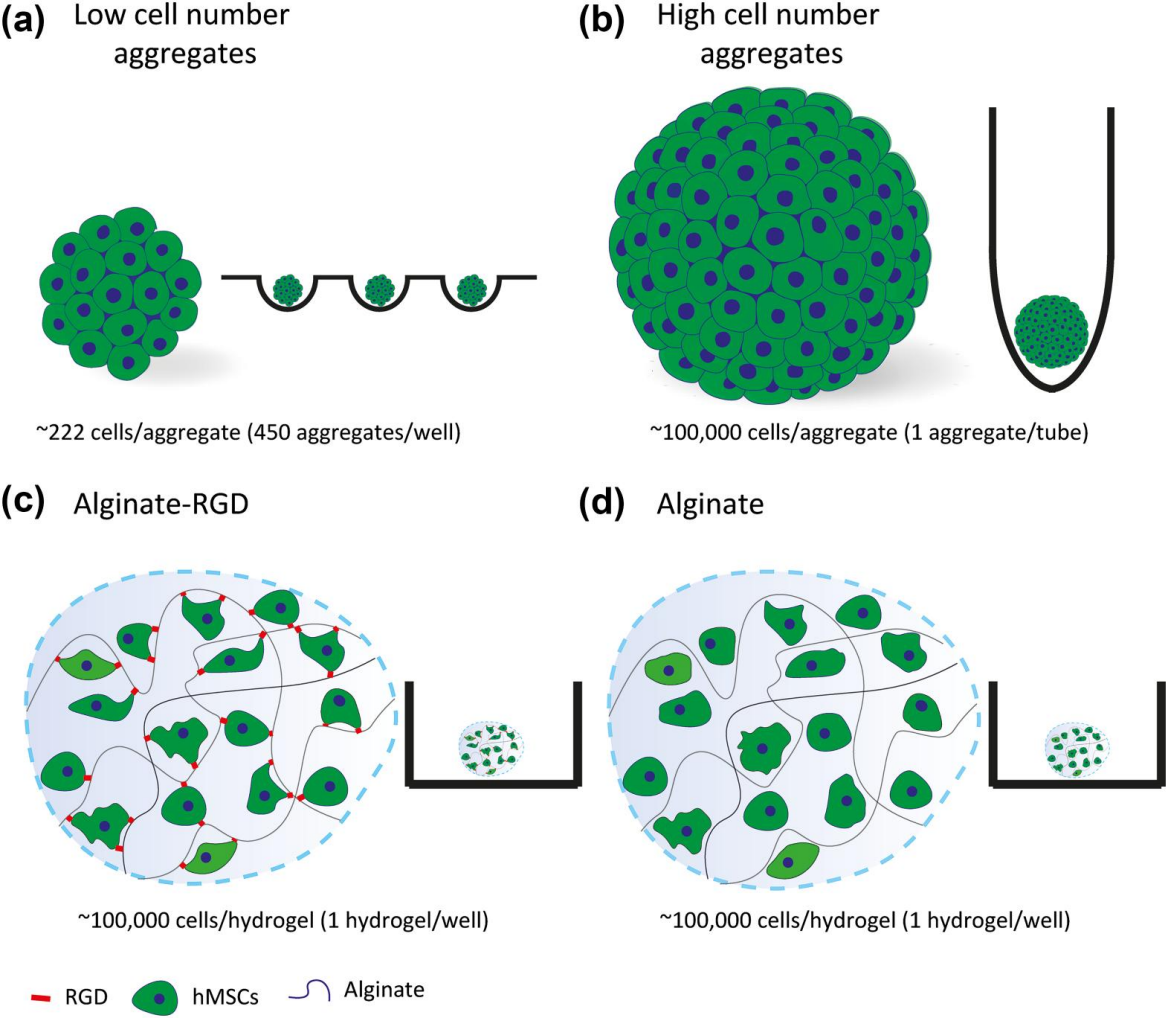
Human mesenchymal stem/stromal cells (hMSCs) in four different cell culture systems. Schematic illustration of the four 3D cell culture systems: (a) hMSCs seeded as low cell number aggregates; (b) hMSCs seeded as a high cell number aggregate; (c) hMSCs encapsulated in alginate hydrogels modified with arginine‐glycine‐aspartic acid (RGD); and (d) hMSCs encapsulated in alginate hydrogels without modification

We sought to answer whether cells are best cultured as aggregates or encapsulated in hydrogels as a cell suspension. In the present study, we look at scaffold‐free self‐assembled aggregates (low and high cell number aggregates), and unaggregated cells encapsulated in alginate hydrogels with and without arginine‐glycine‐aspartic acid (RGD) peptides (Figure 1). We compared these systems based on cell viability, proliferation, and cell cycle analysis over a 7 day culture period in an effort to compare and contrast the different cell culture systems.

## 2 MATERIALS AND METHODS

### 2.1 Cell culture

Bone marrow–derived human MSCs (hMSCs) (PromoCell) were obtained at passage 1 and confirmed free of mycoplasma using the mycoplasma detection kit from BD Biosciences. The cells were maintained in growth medium composed of minimal essential medium (MEM α; Gibco) supplemented with 10% (v/v) fetal bovine serum (Sigma‐Aldrich). The cells were maintained at 37°C in 5% CO_2_ in a humidified incubator and the medium was changed every two days. Upon reaching 80% confluence, cells were detached by incubating with 0.05% trypsin‐ethylenediaminetetraacetic acid (EDTA) (Thermo Fisher Scientific) and re‐plated for continuous passage. The cells were used at passage five for all experiments.

### 2.2 Microwell formation

Agarose microwell arrays were prepared as previously described (Vrij et al., 2016). Briefly, 3% ultra pure agarose solution (Invitrogen) was cast onto a poly(dimethylsiloxane) stamp with microstructures to imprint microwells, de‐molded upon solidification, cut to size, and inserted into 12‐well plates. Each well of the microwell array contained 450 microwells with a diameter of 400 μm.

### 2.3 Low cell number and high cell number aggregate formation

Human mesenchymal stem/stromal cells aggregates were formed in two different sizes of approximately 222 cells (low cell number) or 100,000 cells (high cell number). To form low cell number hMSC aggregates, 100,000 cells in 400 μl growth medium were seeded into one microwell array. The plate was centrifuged at 300 × *g* for 5 min to allow the cells to settle into the microwells, after which an additional 2 ml of growth medium was added to each well. The cells clustered spontaneously within 24 h. To form a high cell number hMSC aggregate, 100,000 cells in 2 ml of growth medium were seeded into a 15 ml polypropylene conical tube (Greiner Bio‐One). The tube was centrifuged at 300 × *g* for 5 min to allow the cells to settle to the bottom. The cells clustered to form an aggregate within 24 h. For both aggregate cultures, medium was changed every 2 days.

### 2.4 Preparation of RGD‐modified alginate

Food grade alginate (70% GG blocks; kindly provided by FMC Polymers) was purified according to a previously published protocol (Neves et al., 2015). Briefly, the alginate was dissolved overnight in ultrapure water (18 MΩ, Milli‐Q UltraPure Water System, Millipore) at a final concentration of 1% (w/v). After dissolution, 2% (w/v) activated charcoal (Sigma‐Aldrich) was added under agitation for 1 h at ambient temperature. The obtained suspension was then centrifuged for 1 h at 27,000 × *g*. Afterwards, the supernatant was passed through a series of filters (1.2, 0.45, and 0.22 μm porous membranes; VWR) via vacuum filtration and was freeze‐dried and stored at −20°C until further use. The alginate was then modified with the peptide (glycine)‐4‐RGD‐serine‐proline (Genscript) to allow cell adhesion using aqueous carbodiimide (EDC) chemistry. Briefly, as described previously (Rowley et al., 1999), a 1% (w/v) alginate solution was prepared in 0.1 M 2‐(N‐morpholino) ethane sulfonic acid (MES) buffer solution (0.1 M MES buffering salt, 0.3 M NaCl, pH adjusted to 6.5 using 1 M NaOH, Sigma‐Aldrich). N‐hydroxy‐sulfosuccinimide (sulfo‐NHS; Pierce Chemical, 27.40 mg per gram alginate) and 1‐ethyl‐(dimethylaminopropyl)‐carbodiimide (EDC; Sigma‐Aldrich, 48.42 mg per gram alginate), at a molar ratio of 1:2, were sequentially added to the solutions, followed by the addition of 16.70 mg RGD per gram alginate. The solution was stirred for 20 h at ambient temperature and quenched with 18 mg of hydroxylamine hydrochloride (Sigma‐Aldrich) per gram of alginate. The final product was dialyzed (MWCO 3500, Spectra/Por, VWR) against decreasing concentrations of NaCl (7.50, 6.25, 5.00, 3.75, 2.50, 1.25 mg) in 4 L of ultrapure water for 3 days at 4°C, freeze‐dried, and stored at −20°C until use. The RGD concentration was 35 μM, as reported in a previous study (Gomes et al., 2021).

### 2.5 Alginate hydrogel formation

Alginate hydrogels containing hMSCs were made by centrifuging 100,000 cells at 500 × *g* for 5 min and resuspending them in 10 μl of 1% (w/v) alginate (either with or without RGD) in NaCl (0.9% [w/v] in water). The alginate hydrogels were formed by dispensing the 10 μl droplet into a 100 mM CaCl_2_ (Sigma‐Aldrich) bath and allowing it to cross‐link for 5 min. The crosslinking solution was then replaced by growth medium for subsequent culture in non‐adherent cell culture plates (VWR). Phase contrast micrographs of hMSCs encapsulated in alginate hydrogels were taken at days 1 and 14 with a Nikon eclipse TS100 inverted microscope.

### 2.6 DNA quantification

A DNA quantification was conducted on days 1, 7, and 14 using the PicoGreen assay (Thermo Fisher Scientific). After measuring luminescence (for the CellTiter‐Glo assay), the samples were lysed in RLT lysis buffer (Qiagen) and stored at −80°C. Samples were freeze‐thawed thrice to ensure their complete lysis. The samples were diluted 1:100 in a solution of 10 mM Tris‐HCl with 1 mM EDTA (pH 7.5) and were analyzed using the PicoGreen assay on a ClarioStar plate reader (BMG LabTech) with the fluorescence signal (excitation: 492 nm and emission: 520 nm) used to extrapolate the DNA concentration from a standard curve.

### 2.7 Live/dead assay

In order to determine the location of the viable cells, a fluorescence‐based live/dead viability assay was conducted on days 1, 7, and 14. Cell aggregates and the alginate hydrogels were washed with Tris‐buffered saline (TBS) after which they were fully immersed in a solution of 2 μM calcein‐AM ester and 5 μM ethidium homodimer‐1 in α‐MEM without phenol red for 30 min at 37°C before imaging directly. The fluorescence images were acquired on a Nikon E600 inverted microscope.

### 2.8 EdU cell proliferation detection

To assess cell proliferation, 5‐ethynyl‐2'‐deoxyuridine (EdU) staining was conducted using the Click‐iT EdU Alexa Fluor 647 Imaging Kit (Thermo Fisher Scientific), according to the manufacturer's protocol. Human mesenchymal stem/stromal cells were incubated with 50 μM EdU for 48 h before fixation at days 2, 7, and 14. Cell aggregates and the alginate hydrogels were washed twice in TBS with 7.5 mM CaCl_2_, and fixed in 4% (v/v) paraformaldehyde (Sigma‐Aldrich) in TBS/CaCl_2_ for 15 min at ambient temperature. Fixed samples were permeabilized with 0.5% (v/v) Triton X‐100 (VWR) in TBS for 1 h and the incorporated EdU was labeled using a click reaction with Alexa Fluor 647 azide for 30 min according to the manufacturer's protocol. The nuclear DNA was counterstained by 4′,6‐diamidino‐2‐phenylindole (0.1 μg/ml) for 30 min. The fluorescence images were acquired on a Nikon E600 inverted microscope.

### 2.9 Cell cycle analysis

To give more information about proliferation, hMSCs were seeded as aggregates or encapsulated in alginate hydrogels in parallel, and cell cycle analysis was conducted on days 1 and 7. The cell aggregates were washed with phosphate buffered saline (PBS) and incubated with 1 ml of accutase (Thermo Fisher Scientific) for 30 min in a water bath at 37°C and the cells were resuspended vigorously every 10 min. The alginate hydrogels were washed with PBS and incubated with 50 mM EDTA in PBS for 10 min at 37°C. After dissociation of both the cell aggregates and alginate hydrogels, the cells were washed twice with ice‐cold PBS. The cells were centrifuged at 300 × *g* and the PBS was aspirated. Ice‐cold absolute ethanol was added dropwise to the cells while vortexing, in which the cells were fixed overnight at 4°C. Fixed samples were washed twice with PBS, resuspended in PBS, and treated with 10 μg/ml RNase A (Invitrogen) and 40 μg/ml propidium iodide (Sigma‐Aldrich) overnight at 4°C in the dark. DNA content was determined by flow cytometry (BD Accuri C6). At least 10,000 events were acquired by pooling three samples for each experimental condition. The percentage of cells in different phases of the cell cycle was assessed using FlowJo software v10.6.0, and the detection of the G1, S, and G2 peaks was carried out manually. The location of the peaks was fixed in order to have the best fit over all the samples.

### 2.10 CellTiter‐Glo 3D Cell Viability Assay

The number of viable cells was determined using the CellTiter‐Glo 3D Cell Viability Assay (Promega) based on the detection of the presence of adenosine triphosphate in living cells according to the manufacturer's protocol. Briefly, cell aggregates and the alginate hydrogels were transferred to a 96‐well plate with 100 μl of growth medium on days 1, 7, and 14, and 100 μl of CellTiter‐Glo 3D Reagent was added into each well. The plate was then placed on an orbital shaker for 5 min and incubated at ambient temperature for an additional 25 min. The luminescence was measured on a ClarioStar plate reader (BMG LabTech) with an integration time of 1 s.

### 2.11 Statistics

Statistics were determined using one‐way ANOVA with Holm‐Sidak's test for multiple comparisons for DNA content and two‐way ANOVA with Tukey's test for multiple comparisons for cell cycle analysis, with *p* values < 0.05 considered significant. Statistical tests were performed with GraphPad Prism 8.

## 3 RESULTS

### 3.1 Cell‐only aggregates decreased in size over time compared to cells encapsulated in alginate hydrogels

We sought to study scaffold‐free self‐assembled high and low cell number aggregates, as well as cells encapsulated in alginate hydrogels with and without RGD peptides (Figure 1). The RGD peptide was selected because it is an important modifier used in polymers for tissue engineering (Klimek & Ginalska, 2020). Since it can be found in various proteins (e.g., collagens, gelatin, elastin, fibronectin, and laminins) and interacts with both α and β integrins, it can provide adhesion to non‐fouling polymers such as alginate. To aggregate hMSCs, we used agarose microwells for low cell number aggregates and 15 ml polypropylene conical tubes for high cell number aggregates and allowed the cells to self‐aggregate. The cell number for each of the conditions was kept constant at 100,000 cells per condition (Figure 1). Once assembled, the low and high cell number aggregates decreased in size over the first 7 days (*p* < 0.0001; Figure S1a,b). Human mesenchymal stem/stromal cells were also encapsulated in the alginate hydrogels with and without RGD and were examined by phase contrast microscopy. There was no significant difference in the size of the alginate hydrogels over time (Figure S1c,d). They also had a similar appearance and had homogeneously distributed hMSCs after 14 days in culture (Figure S2).

### 3.2 DNA content decreases over time for all four culture systems

To quantify the total amount of DNA present over time, a value directly related to the cell number, we used PicoGreen DNA quantification assay in each of the cell culture systems. To make a relative comparison, we compared the differences between the median of three independent experiments in each of the cell culture systems (each having started with 100,000 cells in total). These data were confirmed using the CellTiter‐Glo 3D Cell Viability Assay (Figure S3).

**FIGURE 2:**
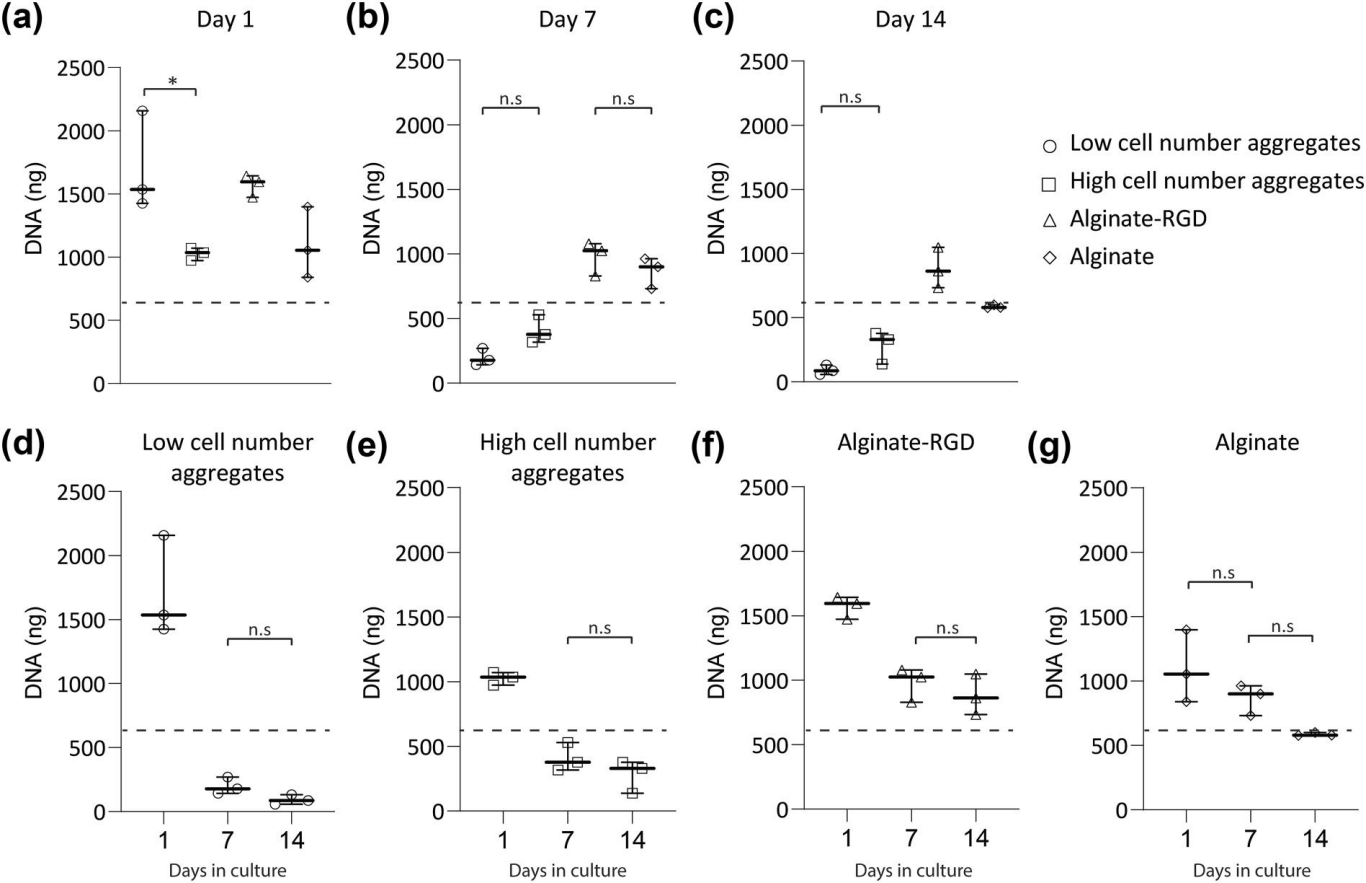
Alginate hydrogels have higher DNA content over time compared to cells in aggregates. Human mesenchymal stem/stromal cells (hMSCs) were seeded in four different cell culture systems: low cell number aggregates, high cell number aggregate, alginate hydrogels without modification and alginate hydrogels modified with arginine‐glycine‐aspartic acid (RGD). The DNA content was analyzed using the PicoGreen assay at (a) day 1; (b) 7; and (c) 14. The same data were also used to compare the different culture systems over time: (d) hMSCs seeded as low cell number aggregates; (e) hMSCs seeded as a high cell number aggregate; (f) hMSCs encapsulated in alginate hydrogels modified with RGD; and (g) hMSCs encapsulated in alginate hydrogels without modification. Data are from three independent experiments. The dotted line indicates the approximate DNA content of 100,000 cells. Statistical significance was determined using one‐way ANOVA with Tukey's test for multiple comparisons. Except for a, all comparisons are statistically significant unless mentioned otherwise; *p < 0.02; n.s: not significant. Data are represented as median with range

On day 1, the DNA content of the different culture systems was not significantly different from each other except when comparing the low and high cell number aggregates (Figure 2a). Namely, the high cell number aggregate had significantly lower DNA content compared to the low cell number aggregates, while they both had similar number of cells while seeding (*p* < 0.04; Figure 2a). Whether cells were encapsulated in alginate or cultured as aggregates had no effect on the DNA content after 1 day. When comparing the measured DNA content to the amount that would come from the 100,000 cells that were seeded (660 ng), both the low cell number aggregates and cells in encapsulated in alginate with RGD had significantly higher DNA content (*p* < 0.003).

After 7 days of culture, a different trend was observed (Figure 2b). In all culture systems, the DNA content had decreased since day 1 (*p* < 0.002) except the cells encapsulated in alginate hydrogels without RGD (Figure 2d–g). When comparing the different culture systems, we observed that the difference in DNA content of low and high cell number aggregates was insignificant, as was the DNA content of cells encapsulated in alginate hydrogels with and without RGD (Figure 2b). The DNA content of the cells encapsulated in alginate hydrogels was significantly higher than cells as aggregates (*p* < 0.002). Compared to the DNA content of 100,000 cells, the cells encapsulated in alginate hydrogels with RGD had significantly higher DNA content, while the low cell number aggregates had significantly lower DNA content (*p* < 0.01; Figure 2b).

After 14 days of culture, there were no statistically significant differences compared to day 7 in all culture systems (Figure 2d–g). However, when comparing the different culture systems we observed that the DNA content was significantly different in all conditions except for low and high cell number aggregates (Figure 2c), where both had significantly lower DNA content compared to cells encapsulated with and without RGD (*p* < 0.02; Figure 2c). Compared to the DNA content of 100,000 cells, only low and high cell number aggregates had significantly lower DNA content (*p* < 0.003; Figure 2c).

### 3.3 Cells remained viable for at least 14 days in culture

Since the different ways of aggregating and encapsulating hMSCs could have an effect on their access to soluble gases and nutrients, we used a live/dead viability assay at days 1, 7, and 14 to determine whether spatial differences could explain the changes in cell number described in Figure 2.

**FIGURE 3:**
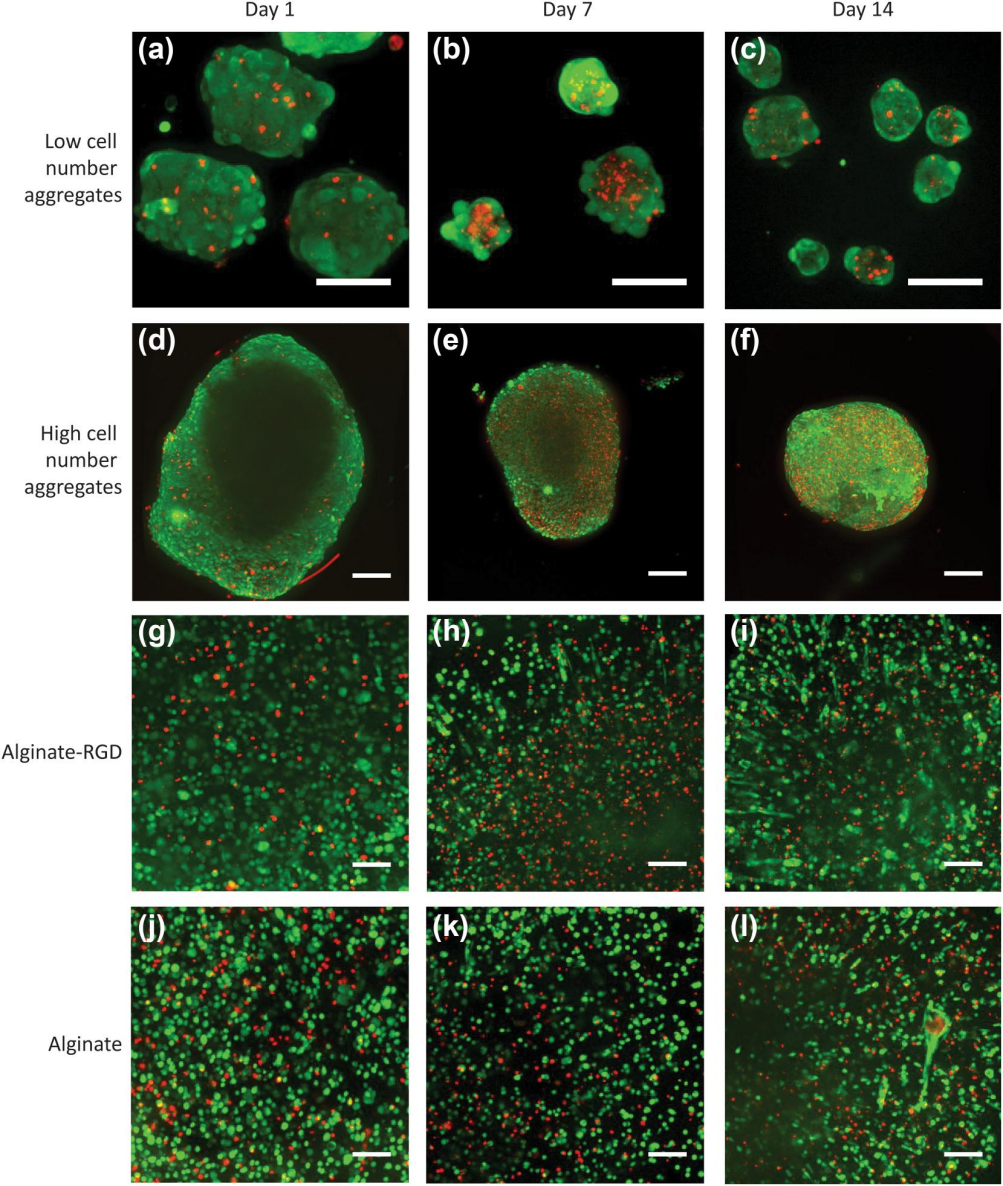
The cell culture systems all maintain viable cells over 14 days in culture. Human mesenchymal stem/stromal cells (hMSCs) were seeded in four different cell culture systems and labeled with calcein‐AM (green; live) and ethidium homodimer‐1 (red; dead) at days 1, 7, and 14. (a–c) Fluorescence micrographs of hMSCs seeded as low cell number aggregates; (d–f) a high cell number aggregate; (g–i) encapsulated in alginate hydrogels modified with arginine‐glycine‐aspartic acid (RGD); and (j–l) encapsulated in alginate hydrogels without modification. Scale bars represent 100 μm. Data are representative of at least three independent experiments with similar results

In the images of low cell number aggregates, we could observe more dead cells, especially in the center of the aggregates, at day 7 (Figure 3b) compared to day 1 (Figure 3a). In the high cell number aggregates, we observed more dead cells at day 7 and 14 compared to day 1 (Figure 3d–f). Similarly, for cells encapsulated in alginate hydrogels, at the periphery, with (Figure 3g–i) or without (Figure 3j–l) RGD, we observed similar numbers and distribution of both live and dead cells at all time points. We observed more dead cells at day 7 for cells encapsulated in alginate with RGD compared to day 1, however for cells encapsulated in alginate without RGD, there was no observable difference in the number of dead cells at all three time points. This is consistent with DNA content results (Figure 2f,g).

### 3.4 The long‐term culture of hMSCs in 3D culture systems decreases proliferation

To understand if the sharp decrease in DNA content in aggregates could be attributed to an increase in cell death or if cell proliferation played a role, we set out to investigate the differences in proliferation rates between the samples. Given that the doubling time of these hMSCs on tissue culture polystyrene was approximately 48 h, we used a 48‐h EdU incubation to detect proliferating cells. The analysis was done at days 2, 7, and 14 on images of whole‐mounted samples.

**FIGURE 4:**
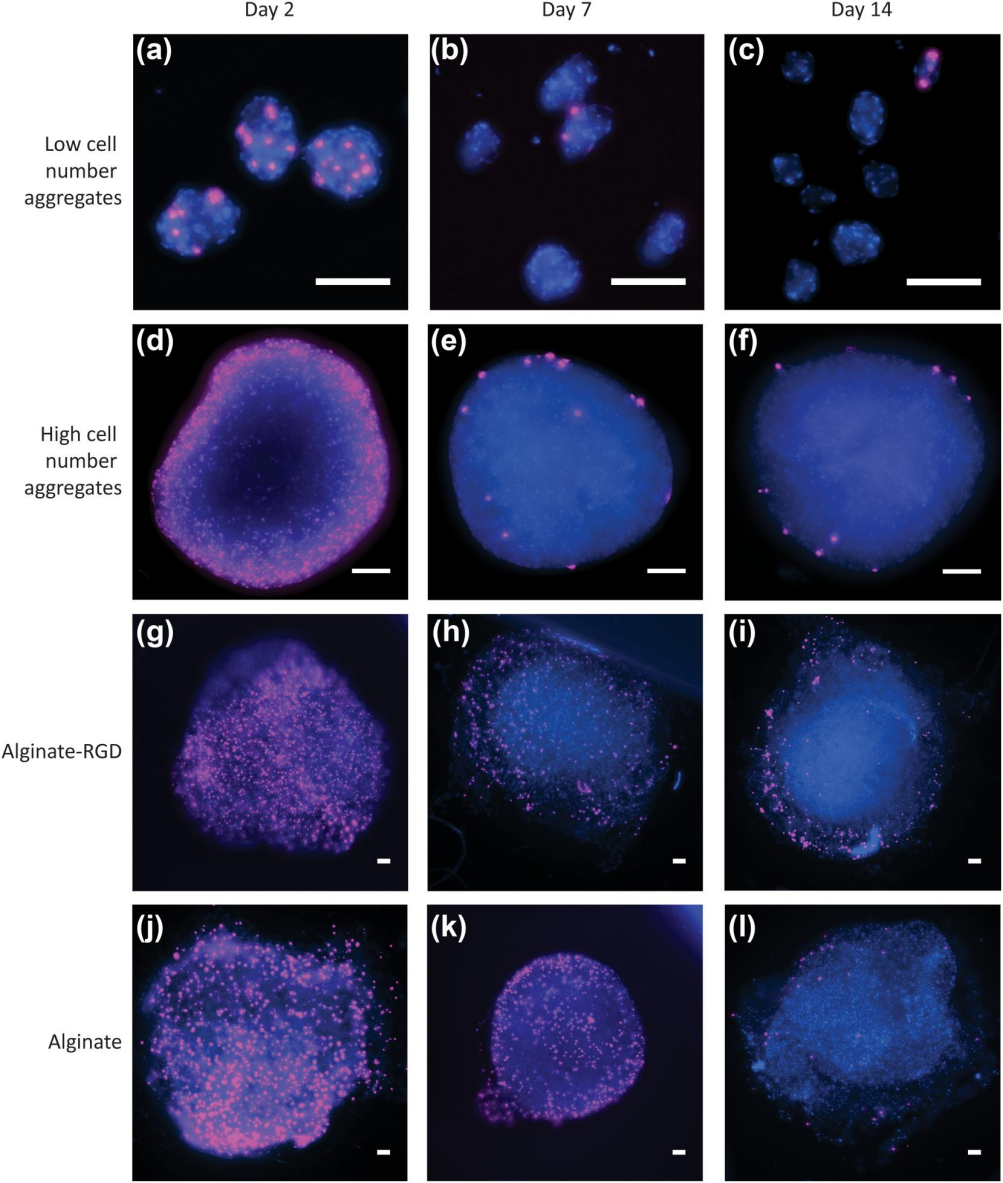
Long‐term culture of human mesenchymal stem/stromal cells (hMSCs) as aggregates and in alginate hydrogels suppresses proliferation. hMSCs were seeded in four different cell culture systems and were subjected to 5‐ethynyl‐2'‐deoxyuridine (pink) for 48 h prior to analysis on days 2, 7 and 14. The samples were counterstained with 4′,6‐diamidino‐2‐phenylindole (DAPI) (blue). (a–c) Fluorescence micrographs depict hMSCs seeded as low cell number aggregates; (d–f) high cell number aggregate; (g–i) encapsulated in alginate hydrogels modified with arginine‐glycine‐aspartic acid (RGD); and (j–l) encapsulated in alginate hydrogels without modification at day 2, 7, and 14. Scale bars represent 100 μm. Data are representative of at least three independent experiments with similar results

In all four cell culture systems, proliferating cells were detected at day 2 (Figure 4). The low cell number aggregates had proliferating cells in 100% the 73 aggregates analyzed on day 2 (Figure 4a), but on days 7 and 14, only 7% of the aggregates analyzed contained proliferating cells (Figure 4b,c). In high cell number aggregates, there were more proliferating cells visible in the periphery of the aggregates on day 2 (Figure 4d), which was noteably diminished by days 7 and 14 (Figure 4e,f). Microscopy limitations prevented us from getting a clearer picture of the center of the large aggregates.

In the alginate hydrogels either with or without RGD, there was no discernible difference between the number of proliferating cells at day 2 (Figure 4g,j). Similar to the aggregates, fewer proliferating cells were observed in the alginate hydrogels at day 7 (Figure 4h,k) and day 14 (Figure 4i,l) compared to day 1. Taken together, these results indicate that the differences noted in DNA content in Figure 2 might be due to differences in both cell death and proliferation rates.

### 3.5 Low cell number aggregates inhibit cell cycle progression

**FIGURE 5:**
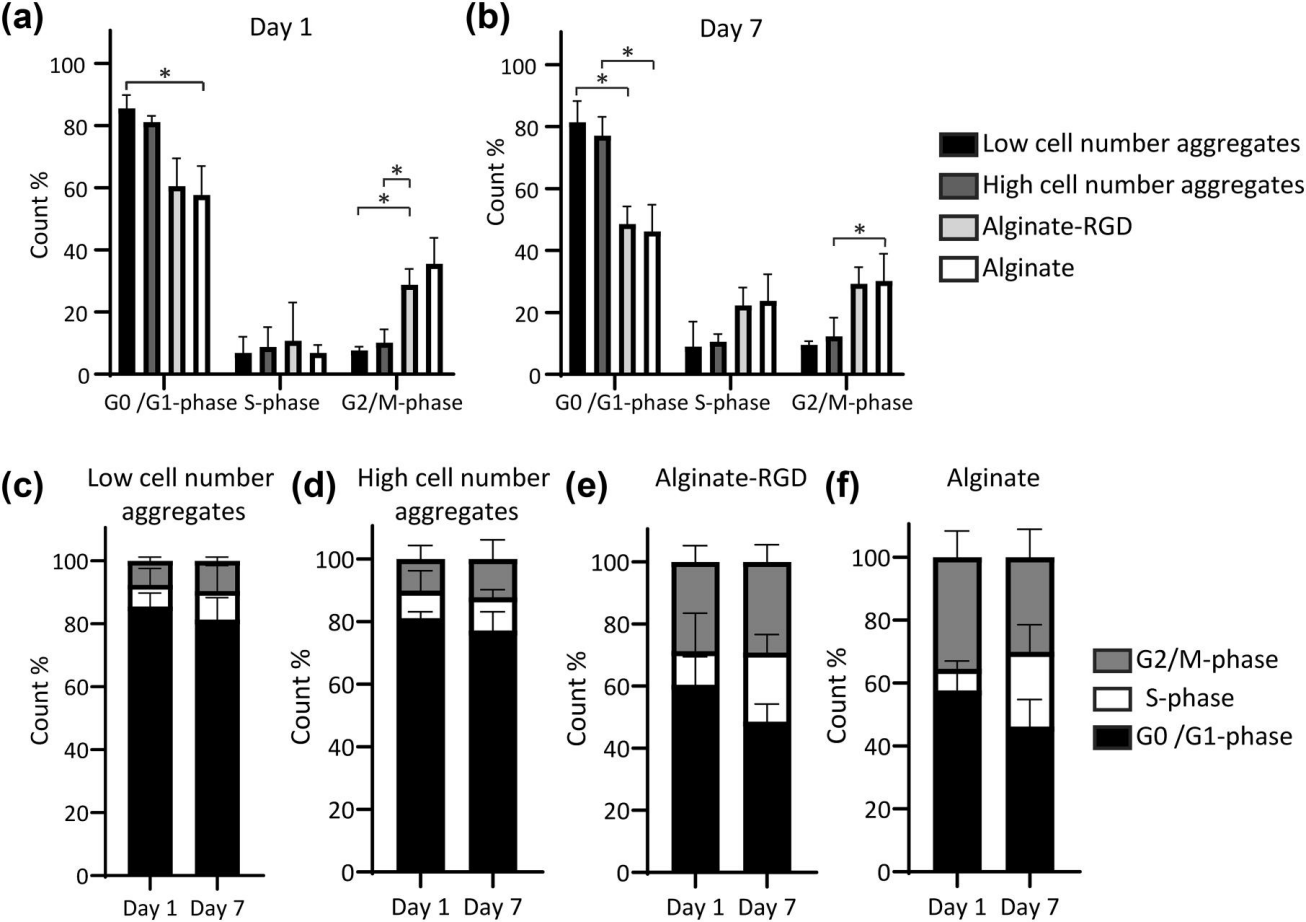
Low cell number aggregates have less cell cycle progression. Human mesenchymal stem/stromal cells (hMSCs) that were seeded in four different cell culture systems and were labeled for cellular DNA content followed by flow cytometry at days 1 and 7. Bar graph represents the quantitative measurement cell cycle phases (G0/G1, S, G2/M) at (a) day 1; (b) 7. The same data were also used to compare the different culture systems over time using stacked bars: hMSCs seeded as (c) low cell number aggregates; (d) a high cell number aggregate; (e) encapsulated in alginate hydrogels modified with arginine‐glycine‐aspartic acid (RGD); and (f) encapsulated in alginate hydrogels without modification. Error bars represent mean ± SD. Data are from three independent experiments. Statistical significance was determined using two‐way ANOVA with Tukey's test for multiple comparisons: *p < 0.04

Having observed a decrease in the number of proliferating cells over time, we wanted to determine how hMSCs were progressing through the cell cycle in the different culture systems. Cell cycle analysis was done using flow cytometry at days 1 and 7 (Figure 5). Overall, we observed no significant differences in the number of cells in the S‐phase at either day 1 and day 7 when we compared the different culture systems (Figure 5a,b), whereas differences were noted in the number of cells in the G_0_/G_1_‐phase and the G_2_/M‐phase.

When observing how cells progressed through the cell cycle at days 1 and 7, there were differences that could be attributed to the culture system. On day 1, the low cell number aggregates had significantly more cells in the G_0_/G_1_‐phase compared to cells in the alginate without RGD (*p* < 0.02; Figure 5a). Furthermore, there were significantly more cells in the G_2_/M‐phase in alginate with RGD compared to the low and high cell number aggregates (*p* < 0.03; Figure 5a). Later, on day 7, there were significantly more cells in the G_0_/G_1_‐phase in low cell number aggregates compared to cells in alginate with RGD (*p* < 0.03; Figure 5b). There were more cells in alginate without RGD in the G_2_/M‐phase compared to in the high cell number aggregates (*p* < 0.02; Figure 5f).

In low cell number aggregates at day 1, we observed 85.5 ± 4.2% cells were in G_0_/G_1_‐phase, 6.7 ± 5.2% in S‐phase, and 7.6 ± 1.1% in G_2_/M‐phase (Figure 5c). On day 7, this was not significantly different, and we measured 81.3 ± 6.9% cells in G_0_/G_1_‐phase, 9.0 ± 8.0% in S‐phase, and 9.5 ± 1.1% in G_2_/M‐phase. This suggests that the low rate of proliferation observed was due to a large number of cells arrested in the G_0_/G_1_‐phase after aggregate formation. In high cell number aggregates at day 1, 81.0 ± 2.0% of cells were in G_0_/G_1_‐phase, 8.1 ± 6.3% in S‐phase, and 10.1 ± 4.2% cells in G_2_/M‐phase (Figure 5d). On day 7, this was not significantly different, and we measured 77.1 ± 5.9% of cells in G_0_/G_1_‐phase, 10.5 ± 2.4% in S‐phase, and 12.2 ± 6.1% in G_2_/M‐phase. Overall, there were no significant differences between low and high cell number aggregates in the cell cycle phases at either time point (*p* > 0.05).

Analyzing the cells encapsulated in alginate with RGD at day 1, 60.4 ± 8.9% were in G_0_/G_1_‐phase, 10.7 ± 12.2% in S‐phase, and 28.7 ± 5.1% cells in G_2_/M‐phase (Figure 5e). On day 7, this was not significantly different, and we measured 48.5 ± 5.6% of cells in G0/G1‐phase, 22.2 ± 5.7% in S‐phase, and 29.2 ± 5.4% in G_2_/M‐phase. For the cells encapsulated in alginate without RGD at day 1, 57.6 ± 9.2% were in G_0_/G_1_‐phase, 6.8 ± 2.5% in S‐phase, and 35.5 ± 8.2% in G_2_/M‐phase (Figure 5f). On day 7, this was not significantly different, and we measured 46.1 ± 8.6% of cells in G_0_/G_1_‐phase, 23.7 ± 8.5% in S‐phase, and 30.1 ± 8.8% in G_2_/M‐phase. There were no significant differences between cells encapsulated in alginate with RGD and without RGD in the cell cycle phases at either time point (*p* > 0.05).

## 4 DISCUSSION

Human mesenchymal stem/stromal cells are an attractive candidate for the development of regenerative therapies, and employing 3D cell culture systems is one possible way to maximize their therapeutic potential (Bartosh et al., 2010). In this study, we were able to compare and contrast the cell number (DNA content), viability, proliferation and cell cycle progression of hMSCs in different 3D culture systems, namely: scaffold‐free self‐assembled aggregates of two sizes (termed high and low cell number aggregates) and cells encapsulated in alginate with and without RGD functionalization. Overall, we noted changes in the DNA content and proliferation, while the cell cycle progression of the hMSCs in the different culture systems remained unchanged over 7 days in culture.

A quantitative DNA assay revealed a decrease in DNA content in all culture systems over time (Figure 2). This decrease was more pronounced when cells were cultured in aggregates than when they were encapsulated in alginate. Low cell number aggregates ended with the lowest DNA content at day 14. A similar outcome was observed in a study that showed that hMSC aggregates undergo apoptosis unless they get appropriate signals for differentiation (Kelm et al., 2012). The overall decrease in the size of low cell number aggregates was also consistent with the decrease in DNA content.

Studies have shown that aggregation can keep hMSCs viable for longer periods compared to adherent cultures (Bartosh et al., 2010; Frith et al., 2010). However, both studies attributed their outcomes to the use of dynamic 3D culture methods, which is in contrast to the static culture techniques used in this study. Here, we found that aggregation itself (assessed at day 1) did not reduce the cell numbers, but it nonetheless appeared that the cells lacked some survival cues (Figure 2). The decrease in DNA content we measured may be the result of poor nutrient and oxygen diffusion to all cells present in aggregates due to crowdedness. In contrast, culturing in the alginate hydrogels may resolve this issue by providing more space between cells, allowing for more nutrients and oxygen diffusion, and thereby higher DNA content. In fact, researchers have shown that glucose, thymidine and proteins such as insulin growth factor‐1, growth hormone and bovine serum albumin were able to diffuse inside alginate hydrogels (Enobakhare et al., 2006). For most of these molecules, 4 h were enough to reach 80% equilibrium. Based on these studies, we conclude that our 1% alginate gels harbor similar diffusion kinetics.

To further investigate the differences in cell number and to obtain spatial information about the location of dead cells, we performed a live/dead assay (Figure 3). We observed an increase in cell death on day 7 compared to day 1 in all culture systems, which was consistent with our DNA content result as well as other studies that have shown increase in cell death due to apoptosis when MSCs are cultured as aggregates under static conditions (Deynoux et al., 2020). However, it seemed that the differences in cell death could not be attributed to their spatial distribution, as a necrotic core was observed in small cell number aggregates but not in high cell number aggregates or in either alginate hydrogel systems.

To explain why aggregates had lower DNA content, we hypothesized there was an imbalance between cell death and proliferation. We demonstrated that cells proliferated in all samples until day 2, but this decreased after one week in culture and remained stable until two weeks (Figure 4). Cells at the periphery of aggregates and hydrogels were more proliferative than cells in the centers, which correlates to previous research findings stating that proliferation occurs when cells have access to appropriate nutrients, correct signaling molecules, and sufficient oxygen (Edmondson et al., 2014; Ullah et al., 2015). Overall, the aggregates seem to promote less proliferation compared to the alginate hydrogels. This low, but present, degree of proliferation is likely the reason why alginate hydrogels show better maintenance of DNA content over time. The overall decrease in the size of low cell number aggregates is also consistent with the decrease in cell number, the decrease in the number of proliferating cells, and cell death. Past studies have suggested that this could be due to cell compaction (Tsai et al., 2015), which was not observed in our study.

To further examine proliferation, we looked at how cells progress through the cell cycle and found no significant differences between the different time points for the different culture systems (Figure 5). This was in contrast to what we observed using the EdU proliferation assay, but was in agreement with a recent finding (Deynoux et al., 2020). One explanation for this difference could be that we added EdU to the cells immediately after seeding and encapsulation but before aggregation. Since hMSCs take approximately 24 h to form aggregates, the EdU incorporation into proliferating hMSCs occurred when they were still in a single cell suspension. This may also explain why the DNA content at day 1 increased compared to the amount we would expect from seeding 100,000 cells.

Overall, no significant differences were observed between cells encapsulated in alginate with and without RGD, which might be explained by the relatively low amount of RGD peptide incorporated into the hydrogel. A previous study has shown that increasing the density of RGD grafted onto alginate hydrogels led to more adhesion, cell spreading, and proliferation, while small amounts of RGD induced myoblasts to acquire a more rounded morphology (Klimek & Ginalska, 2020). In this study, we may have not reached a sufficiently high number of grafted peptides and, therefore, did not observe a significant difference between alginate with and without RGD. In addition, the alginate concentration may have promoted less spreading, even in the presence of the RGD peptide. Other researchers have reported that 0.5% alginate–RGD induces little to no spreading of MSCs and ADSCs, especially compared to 2% alginate–RGD (Dumbleton et al., 2016). Future studies with different concentrations of RGD and other relevant adhesion motifs should be conducted to understand how these peptides influence the outcome of 3D hMSC culture systems. For instance, comparing the performances of alginate hydrogels grafted with GHK (derived from osteonectin), GFOGER (collagen type I), and IKVAV (laminin), amongst others, may allow us to better design an ideal 3D culture system (Formo et al., 2015; Klontzas et al., 2019; Neves et al., 2020; Stephan et al., 2015).

In summary, the research performed here assessed four different 3D cell culture systems with two different variants in each to see how they influence the behavior of hMSCs: scaffold‐free self‐assembled aggregates of two sizes and cells encapsulated in alginate with and without RGD functionalization. From our measurements, we observed that the alginate constructs (both with and without the RGD peptide), appear to better sustain the cells over time. In conclusion, this study underlines the notion that alginate hydrogels might be able to keep hMSCs viable for a longer period compared to cell aggregates.

## CONFLICT OF INTEREST

The authors declare no conflict of interest.

## AUTHOR CONTRIBUTIONS

Fiona R. Passanha, David B. Gomes, Lorenzo Moroni, Matthew B. Baker, and Vanessa L. S. LaPointe designed the research; Fiona R. Passanha, David B. Gomes, Justyna Piotrowska, and PRO3011 Students performed the experiments; Fiona R. Passanha, David B. Gomes, Matthew B. Baker, and Vanessa L. S. LaPointe analyzed data; and Fiona R. Passanha, David B. Gomes, Lorenzo Moroni, Matthew B. Baker, and Vanessa L. S. LaPointe wrote the paper.

## Supporting information

Supplementary Material

## Data Availability

The data that support the findings of this study are available from the corresponding author upon reasonable request.
