# Supplementary material for: A comparative study of mesenchymal stem cells cultured as cell‐only aggregates and in encapsulated hydrogels
Source: J Tissue Eng Regen Med. 2021 Oct 22;16(1):14–25. doi: 10.1002/term.3257 (PMC9297862; doi:10.1002/term.3257)
Supplement: Supplementary file 1 — Supplementary Material [file TERM-16-14-s001.docx]

**Supplementary Figure 1**

**
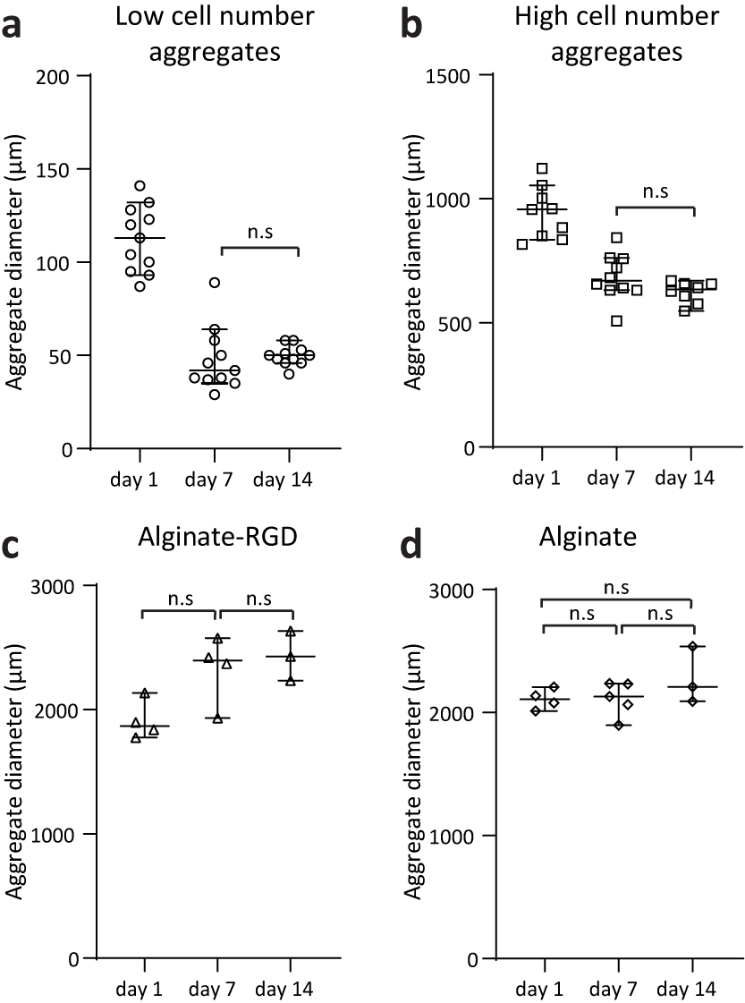
**

**Supplementary Figure 1: Aggregates decreased in size over time for both low and high cell number aggregates.**

The diameter of the hMSCs seeded as low cell number aggregates (a), as high cell number aggregate (b), in alginate hydrogels modified with RGD (c), and in alginate hydrogels without modification (d) was measured from fluorescence micrographs of hMSCs stained with calcein-AM from the live/dead assay and DAPI from the EdU assay at days 1, 7 and 14. The diameters of the aggregates and alginate hydrogels were measured using ImageJ 1,52b software. Data are from three independent experiments. All comparisons are statistically significant (p < 0.05) unless mentioned otherwise; n.s: not significant; error bars represent median ± 95% CI.

**Supplementary Figure 2**

**b**

**a**

Alginate-RGD Alginate


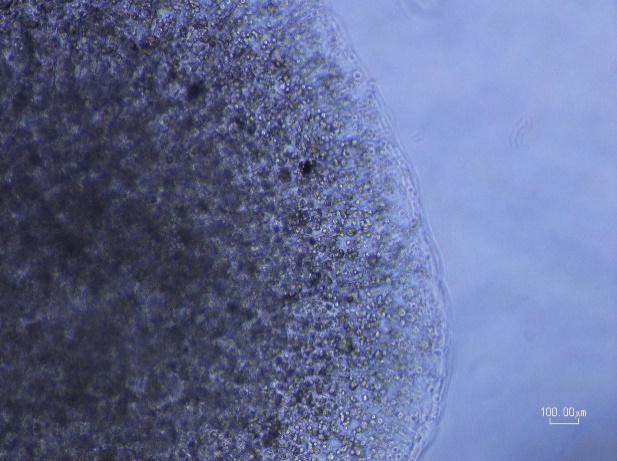

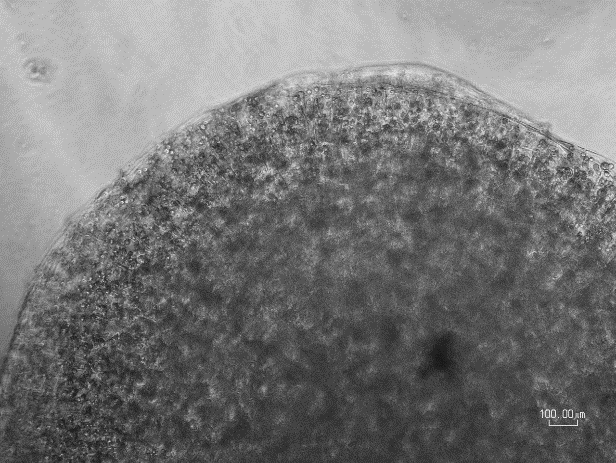


**Supplementary Figure 2: Cells encapsulated in alginate hydrogels with and without RGD modification look visually similar.**

Phase contrast micrographs of hMSCs encapsulated in alginate hydrogels with RGD modification (a) and without RGD modification (b) after 14 days in culture. Scale bars represent 100 μm.

**Supplementary Figure 3**

**
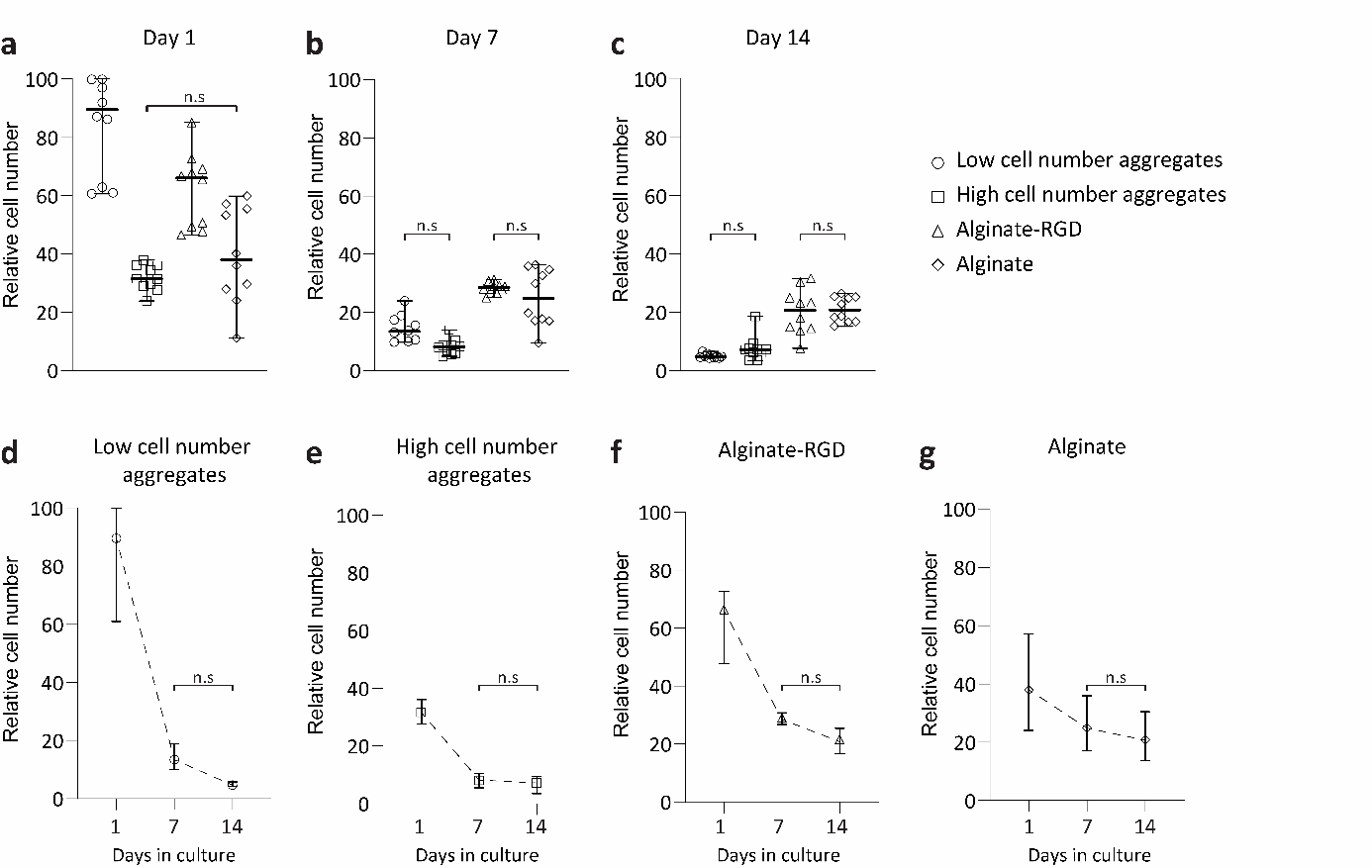
**

**Supplementary Figure 3: Alginate hydrogels have higher viability over time compared to cells as aggregates.**

HMSCs were seeded in four different cell culture systems: low cell number aggregates, high cell number aggregate, alginate hydrogels without modification, and alginate hydrogels modified with RGD. The number of viable cells was evaluated using the CellTiter-Glo 3D cell viability assay at days 1, 7, and 14 (a–c). The same data were also used to compare the different culture systems over time: hMSCs seeded as low cell number aggregates (d), as a high cell number aggregate (e), encapsulated in alginate hydrogels modified with RGD (f), and encapsulated in alginate hydrogels without modification (g). Data are from 10 independent experiments. Statistical significance was determined using one-way ANOVA with Tukey’s test for multiple comparisons and all comparisons are statistically significant (*p* < 0.03) unless mentioned otherwise; n.s: not significant. (a–c) data are represented as median with range. (d–g) error bars represent median ± 95% CI.
